# Associations of Pre-Diagnostic Serum Levels of Total Bilirubin and Albumin With Lung Cancer Risk: Results From the Southern Community Cohort Study

**DOI:** 10.3389/fonc.2022.895479

**Published:** 2022-06-23

**Authors:** Hyung-Suk Yoon, Xiao-Ou Shu, Chris Shidal, Jie Wu, William J. Blot, Wei Zheng, Qiuyin Cai

**Affiliations:** Division of Epidemiology, Department of Medicine, Vanderbilt Epidemiology Center, Vanderbilt-Ingram Cancer Center, Vanderbilt University School of Medicine, Nashville, TN, United States

**Keywords:** lung cancer, total bilirubin (TB), albumin (ALB), smoking, Southern Community Cohort Study, low-income populations, African Americans

## Abstract

**Background:**

Previous studies conducted among European and Asian decedents reported inverse associations of serum total bilirubin and albumin with lung cancer risk. Yet, no study has been conducted among African Americans or low-income European Americans.

**Methods:**

This study included 522 incident lung cancer cases and 979 matched controls nested in the Southern Community Cohort Study, a cohort of predominantly low-income African and European Americans. Serum levels of total bilirubin and albumin, collected up to 11 years prior to case diagnoses, were measured by a clinical chemistry analyzer. Conditional logistic regression models were applied to evaluate the associations of total bilirubin and albumin with lung cancer risk.

**Results:**

Overall, serum levels of total bilirubin (OR_T3 vs. T1_ = 0.96, 95% CI: 0.66-1.39) were not significantly associated with lung cancer risk. However, higher levels of serum total bilirubin were significantly associated with decreased risk of lung cancer among participants who were diagnosed within two years following sample collection (OR_T3 vs. T1_ = 0.36, 95% CI: 0.15-0.87) and among former/never smokers (OR_T3 vs. T1_ = 0.54, 95% CI: 0.32-0.93). Serum levels of albumin were significantly associated with decreased risk of lung cancer overall (OR_T3 vs. T1_ = 0.70, 95% CI: 0.50-0.98) and among African Americans (OR_T3 vs. T1_ = 0.62, 95% CI: 0.41-0.96), but not among European Americans.

**Conclusion:**

Our results indicate that in a low-income African American and European American population, serum levels of total bilirubin may be related to lung cancer progression and differ by smoking status. Meanwhile, the association of serum albumin levels with lung cancer risk may differ by race. Further studies are warranted to confirm these results.

## Introduction

Lung cancer is the third most common type of cancer, and its incidence varies by race/ethnicity and geographical location (1, 2). Cigarette smoking is the primary risk factor for lung cancer, but the mechanisms of lung carcinogenesis are still not well understood (3).

Antioxidants are free radical scavengers that can prevent damage from excessive reactive oxygen species (4) and may act as protective factors in carcinogenic progress (5). Bilirubin and albumin have attracted increasing attention due to their potent antioxidant properties, which may help prevent cancer by reducing inflammation and tumor cell proliferation (6–10). Bilirubin is produced as a byproduct of heme degradation and mostly transported in blood tightly bound to albumin, but a very small fraction of bilirubin remains unbound, *i.e.*, free bilirubin, which has biological effects (11, 12). Several prospective studies investigating the associations of total bilirubin and albumin levels with lung cancer risk have been conducted (13–16), and a meta-analysis indicated an inverse association between total bilirubin levels and risk of lung cancer (17). However, this epidemiological evidence came mainly from studies conducted among European and Asian descendants. No study has been conducted among African Americans or socioeconomically disadvantaged populations who were experiencing elevated lung cancer rates (18).

In this study, we investigated the associations of pre-diagnostic serum levels of total bilirubin and albumin with lung cancer risk in the Southern Community Cohort Study (SCCS), a cohort study comprised of a large proportion of low-income African and European Americans. We further investigated whether the associations were modified by race, smoking status, and histological subtype.

## Methods

### Study Population

The SCCS is a prospective cohort study designed to investigate the underlying causes of racial disparities in health outcomes. Detailed information on the SCCS is described in previous literature (19, 20). All study participants signed written informed consent before enrollment into the SCCS. Approximately 86% of the participants were recruited at community health centers (CHCs) that provide primary health care services for the primarily low-income and uninsured population. Trained interviewers conducted computer-assisted personal interviews to collect baseline data on demographic characteristics and potential health risk factors, including medical history, dietary habits, physical activity, smoking, alcohol consumption, and anthropometrics. Blood samples were collected for nearly half of CHC-enrolled participants and stored at our Vanderbilt freezer facilities.

A total of 522 incident lung cancer cases (defined by the International Classification of Diseases, 10^th^ Revision: ICD-10, C340–C349) with stored blood specimens were ascertained *via* linkage with state cancer registries operating in the 12-state study area and/or from the National Death Index mortality records, by November 2016, and included in the current study. Controls (N=979) with stored blood samples were randomly selected from cancer-free SCCS participants and individually matched to cases at a 2:1 or 1:1 ratio on age ( ± 2 years), race (African American or European American), and sex, as well as the date ( ± 6 months) and site (CHC) of study enrollment, and thus, the date of blood collection. Completely de-identified data were available for the current analysis.

### Laboratory Assays

Blood samples were provided by SCCS participants during enrollment at CHCs. After a blood draw, samples were immediately refrigerated and shipped cold overnight to the Vanderbilt Epidemiology Center’s Molecular Epidemiology Laboratory. Serum samples were isolated and stored at -80°C. An aliquot of serum samples was sent to the testing lab in dry ice for biomarker analyses. For samples included in the current study, comprehensive metabolic panels, including total bilirubin and albumin levels, were measured using the Beckman Coulter clinical chemistry analyzer (DXC 600) at the core research laboratory of the University of Washington’s Kidney Research Institute. Quality control samples (3%) were included in the assays. The intra- and inter-assay coefficients of variation were 0.9% and 1.9%, respectively, for albumin; and 12.7% and 13.4%, respectively, for total bilirubin. Serum samples from each case-control set were analyzed in the same batch and adjacently to eliminate between-assay variability. The laboratory staff was blinded to the case-control status of serum samples and the identity of quality control samples included in the study.

### Statistical Analysis

This study included 522 lung cancer incident cases and 979 matched controls, which consisted of African Americans (334 lung cancer cases and 629 matched controls) and European Americans (188 lung cancer cases and 350 matched controls). Baseline characteristics between lung cancer cases and matched controls were compared by the chi-squared test (for categorical variables) or the student *t*-test (for continuous variables). Serum levels of total bilirubin and albumin were categorized into race-sex specific tertiles among controls. We also categorized serum levels of total bilirubin and albumin into tertiles among all controls, and the results were similar to those using race-sex specific tertiles. Thus, we present results using race-sex specific tertiles among controls. In addition, we further calculated the total bilirubin/albumin ratio grouped into race-sex specific tertiles. For this procedure, total bilirubin (mg/dL) and albumin (g/dL) were converted to µmol/L. We conducted conditional logistic regression analyses to investigate the associations between tertiles of total bilirubin and albumin and lung cancer risk after adjusting for potential confounders, including baseline age, smoking status (current vs. former vs. never), pack-years (<30 vs. ≥30), alcohol consumption (heavy vs. moderate vs. nondrinker), education (less than 11 years vs. completed high school vs. vocational/technical school vs. university degree or higher), household income (<$15,000 vs. $15,000-$24,999 vs. ≥$25,000), self-report of ever having been diagnosed with chronic obstructive pulmonary disease (COPD; no vs. yes), and body mass index (BMI; ≥ 30 kg/m^2^ vs. 29.9-25.0 kg/m^2^ vs. <25 kg/m^2^). Stratified analyses were performed to investigate modifications by race (African American vs. European American) and the time between sample collection and lung cancer diagnosis (≤2 years vs. >2 years). We further conducted stratified analyses by smoking status and lung cancer histological subtype (adenocarcinoma, squamous cell, and small cell lung cancer). Interactions were evaluated by the likelihood ratio test. All statistical analyses were conducted using SAS 9.4 (SAS Institute, Cary, NC, USA), with *p*-values of less than 0.05 being considered statistically significant.

## Results

Baseline characteristics of the study population are presented in **Table 1**. Lung cancer cases had a higher proportion of current smoking (74.3%) and higher pack-years (30.5 pack-years) than matched controls (46.5% and 19.2 pack-years, respectively). Compared with controls, lung cancer cases were more likely to be less educated, earn less income, be alcohol drinkers, have lower BMI, and have a history of COPD (**Table 1**).

**Table 1:** Baseline characteristics of study population, SCCS.

	Cases	Controls	*p*-value^†^
	(N = 522)	(N = 979)	
**Age (mean ± SD)**	56.4 **±** 9.0	56.3 **±** 9.0	0.77
**Race [N (%)]**			
African Americans	334 (64.0)	629 (64.2)	0.92
European Americans	188 (36.0)	350 (35.8)	
**Gender [N (%)]**			
Men	289 (55.4)	540 (55.2)	0.94
Women	233 (44.6)	439 (44.8)	
**Smoking Status [N (%)]**			
Current	388 (74.3)	455 (46.5)	< 0.01
Former	104 (19.9)	240 (24.5)	
Never	30 (5.7)	284 (29.0)	
**Pack-years****^a^** **[median (IQR)]**	30.5 (17.6-48.0)	19.2 (10.0-36.0)	< 0.01
**Alcohol consumption [N (%)]**			
Heavy	125 (24.0)	162 (16.5)	< 0.01
Moderate^b^	185 (35.4)	341 (34.8)	
Nondrinker	212 (40.6)	476 (48.6)	
**Education [N (%)]**			
Less than 11 years	247 (47.3)	388 (39.6)	< 0.01
Completed high school	173 (33.1)	345 (35.2)	
Vocational/technical school	88 (16.9)	171 (17.5)	
University degree or higher	14 (2.7)	75 (7.7)	
**Household income [N (%)]**			
< $15,000	377 (72.2)	624 (63.7)	< 0.01
$15,000 - $24,999	96 (18.4)	220 (22.5)	
≥ $25,000	49 (9.4)	135 (13.8)	
**Self-report of COPD****^c^** **[N (%)]**			
No	432 (82.8)	885 (90.4)	< 0.01
Yes	90 (17.2)	94 (9.6)	
**BMI [kg/m^2^, median (IQR)]**	25.8 (22.3-30.0)	28.2 (24.5-33.1)	< 0.01
**Total Bilirubin [mg/dL, mean (SD)]**	0.41 (0.22)	0.45 (0.32)	< 0.01
**Albumin [g/dL, mean (SD)]**	4.28 (0.37)	4.31 (0.36)	0.08

^†^Estimated by the student t-test procedure for continuous variables and chi-square test for categorical variables.

a Included former/current smoker.

b Defined as > 0 but ≤ 2 drink/day for men or ≤1 drink/day for women.

c Ever diagnosed with emphysema or chronic bronchitis.

Odds ratios (ORs) for lung cancer risk associated with serum levels of total bilirubin and albumin are presented in **Table 2** and **Supplementary Figure 1**. Overall, serum levels of total bilirubin (OR_T3 vs. T1_ = 0.96, 95% CI: 0.66-1.39) were not significantly associated with the risk of lung cancer. When the analyses were conducted separately for African Americans and European Americans, we observed a marginally significant interaction between race and serum total bilirubin levels (*p* interaction=0.05). Higher serum levels of albumin were significantly associated with decreased risk of lung cancer (OR_T3 vs. T1_ = 0.70, 95% CI: 0.50-0.98). The inverse association remained significant among African Americans (OR_T3 vs. T1_ = 0.62, 95% CI: 0.41-0.96) but not among European Americans (OR_T3 vs. T1_ = 0.73, 95% CI: 0.41-1.31) (**Table 2**). We also evaluated the associations of lung cancer risk with the total bilirubin/albumin ratio, a surrogate measurement of free bilirubin. As shown in **Supplementary Table 1**, no significant associations were found between the total bilirubin/albumin ratio and lung cancer risk, regardless of race.

**Table 2:** Association of serum levels of total bilirubin and albumin with lung cancer risk.

	Median^a^	Cases	Controls	OR (95%CI)^†^	OR (95%CI)^‡^
**Total Bilirubin (mg/dL)** ^b^					
**Total population**		**N=522**	**N=979**		
T1	0.20	162	287	1.00 (Ref.)	1.00 (Ref.)
T2	0.38	165	294	1.16 (0.82-1.63)	1.09 (0.76-1.56)
T3	0.60	195	398	1.07 (0.75-1.52)	0.96 (0.66-1.39)
*p* trend				0.85	0.71
**African Americans**		**N=334**	**N=629**		
T1	0.20	91	179	1.00 (Ref.)	1.00 (Ref.)
T2	0.37	128	214	1.48 (0.98-2.24)	1.49 (0.95-2.33)
T3	0.60	115	236	1.35 (0.86-2.12)	1.22 (0.75-1.98)
*p* trend				0.28	0.61
**European Americans**		**N=188**	**N=350**		
T1	0.20	71	108	1.00 (Ref.)	1.00 (Ref.)
T2	0.40	37	80	0.63 (0.33-1.21)	0.52 (0.26-1.04)
T3	0.60	80	162	0.66 (0.36-1.21)	0.57 (0.30-1.09)
*p* trend				0.25	0.15
***p* interaction****^c^**				0.06	0.05
**Albumin (g/dL)****^b^**					
**Total population**		**N=522**	**N=979**		
T1	3.90	173	283	1.00 (Ref.)	1.00 (Ref.)
T2	4.30	178	339	0.86 (0.62-1.18)	0.88 (0.63-1.23)
T3	4.60	171	357	0.78 (0.56-1.07)	0.70 (0.50-0.98)
*p* trend				0.12	0.04
**African Americans**		**N=334**	**N=629**		
T1	3.90	115	185	1.00 (Ref.)	1.00 (Ref.)
T2	4.30	114	221	0.83 (0.56-1.23)	0.90 (0.60-1.37)
T3	4.60	105	223	0.72 (0.49-1.08)	0.62 (0.41-0.96)
*p* trend				0.11	0.03
**European Americans**		**N=188**	**N=350**		
T1	4.00	58	98	1.00 (Ref.)	1.00 (Ref.)
T2	4.30	64	118	0.88 (0.50-1.55)	0.87 (0.48-1.56)
T3	4.70	66	134	0.85 (0.49-1.45)	0.73 (0.41-1.31)
*p* trend				0.55	0.28
***p* interaction****^c^**				0.89	0.79

Analysis using conditional logistic regression models.

a Median level of each tertile.

b Based on the race- and sex-specific tertiles among controls.

c p interaction between Total Bilirubin/Albumin and race with lung cancer risk.

^†^Adjustment for age, smoking status, and pack-years.

^‡^Adjustment for age, smoking status, pack-years, alcohol consumption, education, household income, history of COPD, and BMI.

We conducted analyses stratified by the time between sample collection and lung cancer diagnosis. Higher serum total bilirubin levels were significantly associated with decreased risk of lung cancer among participants diagnosed ≤2 years after blood collection (OR_T3 vs.T1_ = 0.36, 95% CI: 0.15-0.87; *p* trend=0.03) (**Table 3**). Higher albumin levels were also associated with decreased risk of lung cancer, although not significantly, among participants diagnosed ≤2 years after blood collection (OR_T3 vs.T1_ = 0.57, 95% CI: 0.27-1.18). No significant associations were observed in individuals who were diagnosed >2 years after blood collection for either total bilirubin or albumin (**Table 3**).

**Table 3:** Association of serum levels of total bilirubin and albumin with lung cancer risk by time between blood collection and lung cancer diagnosis.

	Median^a^	Cases	Controls	OR (95%CI)^†^	OR (95%CI)^‡^
≤ 2 years		N=128	N=234		
**Total Bilirubin (mg/dL)** ^b^					
T1	0.20	70	106	1.00 (Ref.)	1.00 (Ref.)
T2	0.37	34	64	0.87 (0.44-1.72)	0.80 (0.38-1.67)
T3	0.60	24	64	0.50 (0.23-1.06)	0.36 (0.15-0.87)
*p* trend				0.07	0.03
**Albumin (g/dL)** ^b^					
T1	4.00	30	52	1.00 (Ref.)	1.00 (Ref.)
T2	4.30	55	77	0.80 (0.38-1.67)	0.70 (0.32-1.52)
T3	4.60	43	105	0.60 (0.30-1.20)	0.57 (0.27-1.18)
*p* trend				0.14	0.14
**> 2 years**		**N=394**	**N=745**		
**Total Bilirubin (mg/dL)****^b^**					
T1	0.20	92	181	1.00 (Ref.)	1.00 (Ref.)
T2	0.40	131	230	1.35 (0.90-2.04)	1.25 (0.81-1.94)
T3	0.60	171	334	1.36 (0.89-2.07)	1.17 (0.75-1.83)
*p* trend				0.22	0.64
**Albumin (g/dL)****^b^**					
T1	3.90	143	231	1.00 (Ref.)	1.00 (Ref.)
T2	4.30	123	262	0.86 (0.60-1.22)	0.88 (0.60-1.28)
T3	4.60	128	252	0.84 (0.58-1.20)	0.74 (0.50-1.09)
*p* trend				0.35	0.12

Analysis using conditional logistic regression model.

a Median level of each tertile.

b Based on the race- and sex-specific tertiles among controls.

^†^Adjustment for age, smoking status, and pack-years.

^‡^Adjustment for age, smoking status, pack-years, alcohol consumption, education, household income, history of COPD, and BMI.

We further conducted analyses by smoking status. Serum total bilirubin levels were associated with decreased risk of lung cancer among former/never smokers (OR_T3 vs.T1_ = 0.54, 95% CI: 0.32-0.93) but not among current smokers (**Table 4**). The associations of serum albumin levels were similar between current smokers and former/never smokers. We found no significant differences across histological types (*i.e.*, adenocarcinoma, squamous cell lung cancer, and small cell lung cancer; data not shown).

**Table 4:** Association of serum levels of total bilirubin and albumin with lung cancer risk by smoking status.

	Current Smokers				Former and Never Smokers			
	Median^a^	Cases	Controls	OR (95%CI)^†^	Median^a^	Cases	Controls	OR (95%CI)^†^
		(N=388)	(N=455)			(N=134)	(N=524)	
**Total Bilirubin (mg/dL)****^b^**								
T1	0.20	117	143	1.00 (Ref.)	0.20	45	144	1.00 (Ref.)
T2	0.39	118	137	1.06 (0.73-1.54)	0.37	47	157	0.90 (0.54-1.51)
T3	0.60	153	175	1.03 (0.73-1.45)	0.60	42	223	0.54 (0.32-0.93)
*p* trend				0.87				0.03
								*p* interaction: 0.19
**Albumin (g/dL)****^b^**								
T1	3.90	128	137	1.00 (Ref.)	4.00	45	146	1.00 (Ref.)
T2	4.30	128	154	0.94 (0.66-1.34)	4.30	50	185	1.03 (0.61-1.72)
T3	4.60	132	164	0.83 (0.58-1.17)	4.60	39	193	0.70 (0.40-1.22)
*p* trend				0.28				0.22
								*p* interaction: 0.60

a Median level of each tertile.

b Based on the race- and sex-specific tertiles among controls.

^†^Adjustment for age, sex, race, smoking status, pack-years, alcohol consumption, education, household income, history of COPD, and BMI.

## Discussion

This study investigated the associations of serum levels of total bilirubin and albumin with lung cancer risk among low-income Americans. Overall, total bilirubin was not associated with lung cancer risk, but higher serum levels of total bilirubin were associated with decreased risk of lung cancer within two years of blood collection and among former/never smokers. In addition, we found that higher levels of albumin were significantly inversely associated with lung cancer risk overall and among African Americans.

Previous studies among European and Asian decedents indicated an inverse association between total bilirubin and lung cancer risk. Horsfall and colleagues reported that a 0.1 mg/dL increase in serum bilirubin levels was associated with a lower risk of lung cancer in both men [incidence rate ratio (IRR)=0.92, 95% CI: 0.89-0.95] and women (IRR=0.89, 95% CI: 0.86-0.93) (9). In the EPIC-Heidelberg cohort study, higher levels of bilirubin showed a marginally significant association with a lower risk of lung cancer [hazard ratio (HR)=0.72, 95% CI: 0.51-1.00]. Wen and colleagues reported that bilirubin was inversely associated with lung cancer risk and mortality in Taiwanese male smokers (14). The Severance Cohort Study in Korea reported an increased risk of lung cancer with bilirubin levels from 0.2 to 0.7 mg/dL in men (HR=2.8, 95% CI: 1.8–4.2), compared with bilirubin levels ≥1.0 mg/dL (15). The authors also observed that one standard deviation (SD) increase in bilirubin was associated with a 30% decreased risk of lung cancer (HR=0.7, 95% CI: 0.5–0.9) among current smokers. A Japanese study using electronic medical records during a median 4.7-year follow-up demonstrated that serum bilirubin levels over 1.2 mg/dL were associated with a reduced risk of lung cancer among men (HR=0.47, 95% CI: 0.27-0.83) compared with ≤1.2 mg/dL (10). A large-scale study from Sweden also reported an inverse association between serum total bilirubin and lung cancer risk (HR_Q4 vs.Q1_ = 0.50, 95% CI: 0.44-0.59) (17): this study showed that higher serum total bilirubin levels decreased the risk of lung cancer among non-smokers (HR=0.45, 95% CI: 0.24-0.86) but not among smokers (HR=0.84, 95% CI: 0.44-1.60). In addition, a meta-analysis of five cohort studies reported that high levels of bilirubin were associated with decreased risk of lung cancer (relative risk=0.69, 95% CI: 0.55-0.86) (17). A study from the UK Biobank reported a potential causal association between serum bilirubin and the incidence of lung cancer by Mendelian Randomization (21); this study suggested that genetically predicted serum bilirubin might protect individuals from exposure hazards to high levels of oxidants associated with cigarette smoking. Unlike previous studies, our study did not find a significant association between total bilirubin levels and lung cancer risk among total study participants. It is worth noting that our study was conducted among low-income African Americans and European Americans. Nevertheless, we found an inverse association of total bilirubin levels with lung cancer risk among cases diagnosed within two years after blood collection. This suggests that total bilirubin may be related to lung cancer progression and could potentially be a lung cancer biomarker. In addition, the inverse association of serum total bilirubin levels was only observed among former/never smokers in our study, indicating that total bilirubin may be a promising biomarker for non-current smokers.

Bilirubin exhibits potent antioxidant effects and has been shown to reduce age-related inflammation and metabolic deterioration in preclinical rodent models (22). The link between inflammation and cancer has long been recognized. Thus, endogenous compounds reducing inflammation have been generally hypothesized to have cancer-preventive properties. Additional mechanisms also indicate a protective role of bilirubin in cancer risk: bilirubin may act as an immuno-modulatory agent and has been shown to suppress CD4 T cell responses (23). CD4-positive T cells, along with their related cytokines, are associated with lung cancer risk (24).

We found that higher albumin levels were associated with decreased risk of lung cancer overall and among African Americans. A previous study reported that higher levels of albumin had a marginally significant association with a lower risk of lung cancer (HR=0.72, 95% CI: 0.51-1.00) (13). Recently, the Kailuan cohort in China showed that pre-diagnostic albumin was inversely associated with lung cancer risk (HR_Q4 vs. Q1_ = 0.70, 95% CI: 0.52-0.95), but the inverse association became insignificant after excluding cases diagnosed within two years of enrollment (25). Another study also reported that one SD increment in albumin was inversely associated with lung cancer risk (HR=0.82, 95% CI: 0.72-0.94) among Koreans (26).

Albumin plays a role as a scavenger and antioxidant (8, 27–29), which may reflect the inflammatory and nutritional status (30). Albumin can be broken down in the cell, but it still provides amino acids for cell proliferation and matrix deposition (28). Low serum albumin levels are generally regarded as an indicator of severe inflammation or malnutrition (28, 31). The inverse correlations of serum albumin levels with C-reactive protein and tumor necrosis factor-α also support the association between serum albumin and inflammation (32–34). Given a strong link of chronic infection and inflammatory microenvironment to lung cancer (35), the inverse associations of serum albumin that we observed in the current study could be biologically plausible. However, future investigations are needed to further explore the biological role of albumin in the development of lung cancer and whether the molecular mechanisms underneath the albumin-inflammation association differ by race/ethnicity or socio-economic status.

The current study has several strengths. First, we conducted a population-based nested case-control study, including African Americans and low-income populations in the US. This could provide a unique insight into the associations of serum total bilirubin and albumin with lung cancer among underserved populations who are at a greater risk for lung cancer. Second, our study used blood samples collected before lung cancer diagnosis and treatment, thus minimizing potential reverse causality. Finally, comprehensive covariate information from the SCCS allowed us to adjust for major confounders. The limitations of our study should also be noted. Larger sample size was necessary for additional stratified analyses by smoking status and lung cancer histological subtypes. Despite the comprehensive adjustments for covariates, we could not completely rule out the influence of residual confounding variables, unmeasured potential confounders, or one-time measurements.

## Conclusion

Our findings show that total bilirubin serum levels were inversely associated with short-term lung cancer incidence, particularly within two years of blood collection. These findings raise the possibility that lung cancer itself and/or its immediate clinical precursors may influence (lower) total bilirubin levels, leading to the appearance of a protective effect of the biomarkers upon lung cancer. However, the absence of associations beyond two years may imply that serum levels of total bilirubin are unrelated to lung cancer risk. In addition, total bilirubin was associated with decreased lung cancer risk among former/never smokers, indicating that total bilirubin may be a promising biomarker for non-current smokers. We also found that serum albumin was inversely associated with lung cancer risk overall and among African Americans. Further studies with a larger sample size are warranted to confirm our findings, especially on effect modification by race/ethnicity and smoking status.

## Data Availability Statement

Data used in the present study can be requested through the Southern Community Cohort Study online request system (https://ors.southerncommunitystudy.org/).

## Ethics Statement

The SCCS protocol was reviewed and approved by the institutional review boards at Vanderbilt University Medical Center and Meharry Medical College. The participants provided their written informed consent to participate in this study.

## Author Contributions

QC conceived of the study. WZ and WB provided study resources. QC and JW conducted laboratory analyses. HSY and CS performed statistical analyses. All authors contributed to interpretation of results. HSY, CS, XS, and QC drafted the manuscript. All authors approved the final version of the manuscript.

## Funding

This work was supported by the National Institutes of Health (T32CA160056, R01CA092447, and U01CA202979). Data collection and sample preparation were performed by the Survey and Biospecimen Shared Resource which is supported in part by the Vanderbilt-Ingram Cancer Center (P30CA68485).

## Conflict of Interest

The authors declare that the research was conducted in the absence of any commercial or financial relationships that could be construed as a potential conflict of interest.

## Publisher’s Note

All claims expressed in this article are solely those of the authors and do not necessarily represent those of their affiliated organizations, or those of the publisher, the editors and the reviewers. Any product that may be evaluated in this article, or claim that may be made by its manufacturer, is not guaranteed or endorsed by the publisher.

## Author Disclaimer

The content is solely the responsibility of the authors and does not necessarily represent the official views of the National Institutes of Health.

## Supplementary Material

Supplementary Figure 1 Association of serum levels of total bilirubin and albumin with lung cancer risk.

Supplementary Table 1 Association of serum levels of total bilirubin/albumin ratio with lung cancer risk.

## Abbreviation

SCCS, Southern Community Cohort Study.

## References

[1] Siegel RL Miller KD Fuchs HE Jemal A. Cancer Statistics, 2021. CA Cancer J Clin (2021) 71:7–33. doi: 10.3322/caac.21654 33433946

[2] American Lung Association. Too Many Cases, Too Many Deaths. In: Lung Cancer in African Americans. (Washington D.C: American Lung Association), (2010).

[3] Ryan BM. Lung Cancer Health Disparities. Carcinogenesis (2018) 39:741–51. doi: 10.1093/carcin/bgy047 PMC5972630 29547922

[4] Pham-Huy LA He H Pham-Huy C. Free Radicals, Antioxidants in Disease and Health. Int J BioMed Sci (2008) 4:89–96. 23675073 PMC3614697

[5] Marnett LJ. Oxyradicals and DNA Damage. Carcinogenesis (2000) 21:361–70. doi: 10.1093/carcin/21.3.361 10688856

[6] Peng YF Goyal H Xu GD. Serum Bilirubin has an Important Role in Multiple Clinical Applications. J Lab Precis Med (2017) 2:82–2. doi: 10.21037/jlpm.2017.09.08

[7] Gupta D Lis CG. Pretreatment Serum Albumin as a Predictor of Cancer Survival: A Systematic Review of the Epidemiological Literature. Nutr J (2010) 9:69. doi: 10.1186/1475-2891-9-69 21176210 PMC3019132

[8] Roche M Rondeau P Singh NR Tarnus E Bourdon E. The Antioxidant Properties of Serum Albumin. FEBS Lett (2008) 582:1783–7. doi: 10.1016/j.febslet.2008.04.057 18474236

[9] Horsfall LJ Rait G Walters K Swallow DM Pereira SP Nazareth I. Serum Bilirubin and Risk of Respiratory Disease and Death. JAMA (2011) 305:691–7. doi: 10.1001/jama.2011.124 21325185

[10] Inoguchi T Nohara Y Nojiri C Nakashima N. Association of Serum Bilirubin Levels With Risk of Cancer Development and Total Death. Sci Rep (2021) 11:13224. doi: 10.1038/s41598-021-92442-2 34168201 PMC8225648

[11] Dudeja V Ferrantella A Fong Y. The Liver. In: Sabiston Textbook of Surgery, 21st ed. Philadelphia, PA: Elsevier (2021).

[12] Adin CA. Bilirubin as a Therapeutic Molecule: Challenges and Opportunities. Antioxidants (2021) 10:1536. doi: 10.3390/antiox10101536 34679671 PMC8532879

[13] Kühn T Sookthai D Graf ME Schübel R Freisling H Johnson T. Albumin, Bilirubin, Uric Acid and Cancer Risk: Results From a Prospective Population-Based Study. Br J Cancer (2017) 117:1572–9. doi: 10.1038/bjc.2017.313 PMC5680462 28898231

[14] Wen CP Zhang F Liang D Wen C Gu J Skinner H. The Ability of Bilirubin in Identifying Smokers With Higher Risk of Lung Cancer: A Large Cohort Study in Conjunction With Global Metabolomic Profiling. Clin Cancer Res (2015) 21:193–200. doi: 10.1158/1078-0432.CCR-14-0748 25336700 PMC4286447

[15] Lim JE Kimm H Jee SH. Combined Effects of Smoking and Bilirubin Levels on the Risk of Lung Cancer in Korea: The Severance Cohort Study. PloS One (2014) 9:e103972. doi: 10.1371/journal.pone.0103972 25100210 PMC4123988

[16] Sprague BL Trentham-Dietz A Klein BEK Klein R Cruickshanks KJ Lee KE. Physical Activity, White Blood Cell Count, and Lung Cancer Risk in a Prospective Cohort Study. Cancer Epidemiol Biomarkers Prev (2008) 17:2714–22. doi: 10.1158/1055-9965.EPI-08-0042 PMC2692679 18843014

[17] Monroy-Iglesias MJ Moss C Beckmann K Hammar N Walldius G Bosco C. Serum Total Bilirubin and Risk of Cancer: A Swedish Cohort Study and Meta-Analysis. Cancers (Basel) (2021) 13:5540. doi: 10.3390/cancers13215540 34771701 PMC8582941

[18] Giaquinto AN Miller KD Tossas KY Winn RA Jemal A Siegel RL. Cancer Statistics for African American/Black People 2022. CA Cancer J Clin (2022) 72(3):202–229. doi: 10.3322/caac.21718 35143040

[19] Signorello LB Hargreaves MK Steinwandel MD Zheng W Cai Q Schlundt DG. Southern Community Cohort Study: Establishing a Cohort to Investigate Health Disparities. J Natl Med Assoc (2005) 97:972–9. PMC2569308 16080667

[20] Signorello LB Hargreaves MK Blot WJ. The Southern Community Cohort Study: Investigating Health Disparities. J Health Care Poor Underserved (2010) 21:26–37. doi: 10.1353/hpu.0.0245 PMC2940058 20173283

[21] Horsfall LJ Burgess S Hall I Nazareth I. Genetically Raised Serum BilirubinLevels and Lung Cancer: A Cohort Study and Mendelian RandomisationUsing UK Biobank.Thorax (2020) 75(11):955–64. doi: 10.1136/thoraxjnl-2020-214756. PMC7569373 32855344

[22] Zelenka J Dvořák A Alán L Zadinová M Haluzík M Vítek L. Hyperbilirubinemia Protects Against Aging-Associated Inflammation and Metabolic Deterioration. Oxid Med Cell Longev (2016) 2016:6190609. doi: 10.1155/2016/6190609 27547293 PMC4983390

[23] Liu Y Stewart KN Bishop E Marek CJ Kluth DC Rees AJ. Unique Expression of Suppressor of Cytokine Signaling 3 Is Essential for Classical Macrophage Activation in Rodents *In Vitro* and *In Vivo* . J Immunol (2008) 180:6270–8. doi: 10.4049/jimmunol.180.9.6270 18424750

[24] Liao C Yu ZB Meng G Wang L Liu QY Chen LT. Association Between Th17-Related Cytokines and Risk of Non-Small Cell Lung Cancer Among Patients With or Without Chronic Obstructive Pulmonary Disease. Cancer (2015) 121 Suppl 17:3122–9. doi: 10.1002/cncr.29369 26331819

[25] Yang Z Zheng Y Wu Z Wen Y Wang G Chen S. Association Between Pre-Diagnostic Serum Albumin and Cancer Risk: Results From a Prospective Population-Based Study. Cancer Med (2021) 10:4054–65. doi: 10.1002/cam4.3937 PMC8209558 34041866

[26] Kim YR Choi CK Lee YH Choi SW Kim HY Shin MH. Association Between Albumin, Total Bilirubin, and Uric Acid Serum Levels and the Risk of Cancer: A Prospective Study in a Korean Population. Yonsei Med J (2021) 62:792–8. doi: 10.3349/ymj.2021.62.9.792 PMC8382725 34427064

[27] Taverna M Marie AL Mira JP Guidet B. Specific Antioxidant Properties of Human Serum Albumin. Ann Intensive Care (2013) 3:4. doi: 10.1186/2110-5820-3-4 23414610 PMC3577569

[28] Soeters PB Wolfe RR Shenkin A. Hypoalbuminemia: Pathogenesis and Clinical Significance. JPEN J Parenter Enteral Nutr (2019) 43:181–93. doi: 10.1002/jpen.1451 PMC7379941 30288759

[29] Merlot AM Kalinowski DS Richardson DR. Unraveling the Mysteries of Serum Albumin-More Than Just a Serum Protein. Front Physiol (2014) 5:299/abstract. doi: 10.3389/fphys.2014.00299 25161624 PMC4129365

[30] Eckart A Struja T Kutz A Baumgartner A Baumgartner T Zurfluh S. Relationship of Nutritional Status, Inflammation, and Serum Albumin Levels During Acute Illness: A Prospective Study. Am J Med (2020) 133:713–722.e7. doi: 10.1016/j.amjmed.2019.10.031 31751531

[31] Kondrup J Allison SP Elia M Vellas B Plauth M Educational and Clinical Practice Committee, European Society of Parenteral and Enteral Nutrition (ESPEN). ESPEN Guidelines for Nutrition Screening 2002. Clin Nutr (2003) 22:415–21. doi: 10.1016/S0261-5614(03)00098-0 12880610

[32] Ishida S Hashimoto I Seike T Abe Y Nakaya Y Nakanishi H. Serum Albumin Levels Correlate With Inflammation Rather Than Nutrition Supply in Burns Patients: A Retrospective Study. J Med Invest (2014) 61:361–8. doi: 10.2152/jmi.61.361 25264055

[33] Ohwada H Nakayama T Kanaya Y Tanaka Y. Serum Albumin Levels and Their Correlates Among Individuals With Motor Disorders at Five Institutions in Japan. Nutr Res Pract (2017) 11:57–63. doi: 10.4162/nrp.2017.11.1.57 28194266 PMC5300948

[34] Oe Y Mochizuki K Miyauchi R Misaki Y Kasezawa N Tohyama K. Plasma TNF-α Is Associated With Inflammation and Nutrition Status in Community-Dwelling Japanese Elderly. J Nutr Sci Vitaminol (Tokyo) (2015) 61:263–9. doi: 10.3177/jnsv.61.263 26226964

[35] Tan Z Xue H Sun Y Zhang C Song Y Qi Y. The Role of Tumor Inflammatory Microenvironment in Lung Cancer. Front Pharmacol (2021) 12:688625. doi: 10.3389/fphar.2021.688625 34079469 PMC8166205

