# Supplementary material for: Associations of Pre-Diagnostic Serum Levels of Total Bilirubin and Albumin With Lung Cancer Risk: Results From the Southern Community Cohort Study
Source: Front Oncol. 2022 Jun 23;12:895479. doi: 10.3389/fonc.2022.895479 (PMC9261263; doi:10.3389/fonc.2022.895479)
Supplement: Supplementary Figure 1 — Association of serum levels of total bilirubin and albumin with lung cancer risk. [file Image_1.pdf]

A. Total Bilirubin (mg/dL)

	Case	Control	OR (95% CI)	*P* trend
**Total population**				
T1	162	287	1.00 (Ref.)	0.71
T2	165	294	1.09 (0.76-1.56)	
T3	195	398	0.96 (0.66-1.39)	
**African Americans**				
T1	91	179	1.00 (Ref.)	0.61
T2	128	214	1.49 (0.95-2.33)	
T3	115	236	1.22 (0.75-1.98)	
**European Americans**				
T1	71	108	1.00 (Ref.)	0.15
T2	37	80	0.52 (0.26-1.04)	
T3	80	162	0.57 (0.30-1.09)	

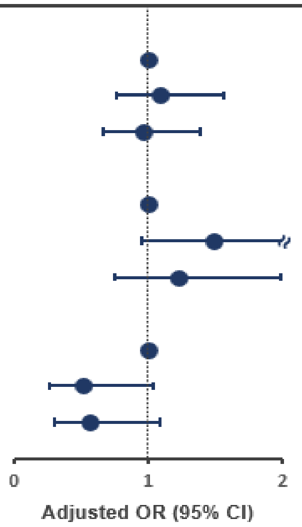

B. Albumin (g/dL)

	Case	Control	OR (95% CI)	*P* trend
**Total population**				
T1	173	283	1.00 (Ref.)	0.04
T2	178	339	0.88 (0.63-1.23)	
T3	171	357	0.70 (0.50-0.98)	
**African Americans**				
T1	115	185	1.00 (Ref.)	0.03
T2	114	221	0.90 (0.60-1.37)	
T3	105	223	0.62 (0.41-0.96)	
**European Americans**				
T1	58	98	1.00 (Ref.)	0.28
T2	64	118	0.87 (0.48-1.56)	
T3	66	134	0.73 (0.41-1.31)	

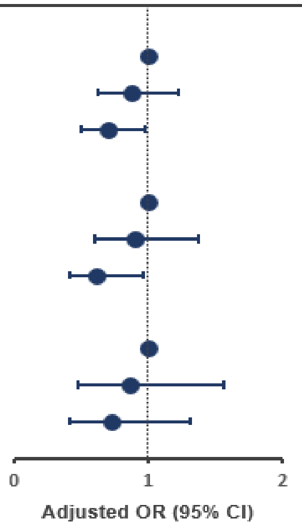

Supplementary Figure 1. Association of Serum levels of Total Bilirubin and Albumin with Lung Cancer Risk

Based on the race- and sex-specific tertiles among controls. Conditional logistic regression models were used to estimate odds ratios and corresponding 95% confidence intervals after adjustment for age, smoking status, pack-years, alcohol consumption, education, household income, history of COPD, and BMI.
