# Supplementary material for: Associations of Pre-Diagnostic Serum Levels of Total Bilirubin and Albumin With Lung Cancer Risk: Results From the Southern Community Cohort Study
Source: Front Oncol. 2022 Jun 23;12:895479. doi: 10.3389/fonc.2022.895479 (PMC9261263; doi:10.3389/fonc.2022.895479)
Supplement: Supplementary Table 1 — Association of serum levels of total bilirubin/albumin ratio with lung cancer risk. [file Table_1.pdf]

Supplementary Table 1. Association of Serum levels of Total Bilirubin/Albumin Ratio[a] with Lung Cancer Risk

	Cases	Controls	OR (95%CI)†	OR (95%CI)‡
**Total population**	**N=522**	**N=979**		
T1	173	317	1.00 (Ref.)	1.00 (Ref.)
T2	186	336	1.16 (0.84-1.59)	1.06 (0.76-1.48)
T3	163	326	1.16 (0.81-1.64)	1.06 (0.73-1.55)
p trend			0.45	0.76
**African Americans**	**N=334**	**N=629**		
T1	100	204	1.00 (Ref.)	1.00 (Ref.)
T2	128	217	1.47 (1.00-2.17)	1.40 (0.92-2.12)
T3	106	208	1.50 (0.97-2.31)	1.34 (0.84-2.14)
p trend			0.08	0.24
**European Americans**	**N=188**	**N=350**		
T1	73	113	1.00 (Ref.)	1.00 (Ref.)
T2	58	119	0.65 (0.36-1.15)	0.53 (0.28-0.99)
T3	57	118	0.63 (0.33-1.18)	0.57 (0.29-1.14)
p trend			0.18	0.16
***p* interaction[b]**			0.15	0.11

Note: Analysis using conditional logistic regression model

[a]Based on the race- and sex-specific tertiles among controls; [b]*p* interaction between Total Bilirubin/Albumin Ratio and race with lung cancer risk

†Adjustment for age, smoking status, and pack-years

‡Adjustment for age, smoking status, pack-years, alcohol consumption, education, household income, history of COPD, and BMI
